# RNA-seq reveals the involvement of key genes for aerobic adaptation in rice

**DOI:** 10.1038/s41598-019-41703-2

**Published:** 2019-03-27

**Authors:** Amol S. Phule, Kalyani M. Barbadikar, Sheshu Madhav Maganti, P. Seguttuvel, D. Subrahmanyam, M. B. B. Prasad Babu, Polumetla A. Kumar

**Affiliations:** 1 Biotechnology Division, ICAR-Indian Institute of Rice Research, Hyderabad, 30 India; 2 Institute of Biotechnology, Professor Jayashankar Telangana State Agricultural University, Hyderabad, 30 India

## Abstract

Adaptation of rice to the aerobic condition is needed to cope with the water scarcity as well as to ensure sustainable yield in future. To understand the molecular mechanisms responsible for aerobic adaptation in rice, we performed RNA-seq analysis of root and shoot i.e. developing panicle tissues at panicle initiation stage in two cultivars adapted to aerobic (CR Dhan 202) and traditional transplanted anaerobic (BPT 5204) conditions. The RNA-seq data emanated from 1.65 billion clean reads with approximately 37 million reads per sample. The number of differentially expressed transcripts was higher in the root than that in the shoot under both aerobic and anaerobic conditions. The transcription factors *viz*. MADS4, MADS5, MADS6, MADS7, MADS15 and transporters involved in sugar (SWEET3A) and nutrient uptake (PHT1;6, MDR-like ABC and vacuolar iron transporter homolog 2) were highly and uniquely expressed in the aerobic adapted cultivar (AAC) CR Dhan 202 under aerobic condition indicating their role in adaptation. The hormones such as ethylene and abscisic acid might be significantly involved in imparting aerobic adaptation. The higher expression of root related genes in the AAC under aerobic conditions suggests the involvement and sensitivity of roots to the water limiting condition. The metabolic activities are also more pronounced in the roots which impart rigorous plant establishment under the aerobic condition. The presence of alternative splice variants in the transcripts *viz*. Tetratrico peptide repeat (TPR) domain containing protein and GOLDEN2-LIKE1 (GLK1) additionally confirms that post transcriptional regulation is also crucial for aerobic adaptation. The QTLs related to root traits and stress tolerance harboring the uniquely expressed genes, which were identified in the present study can be deployed in molecular breeding programs to develop elite, high yielding aerobic rice cultivars.

## Introduction

The changing climate, depleting water resources and increasing population make it imperative to develop next-generation climate smart crop varieties for sustainable agriculture. Rice, a major staple food crop is widely cultivated in diverse production systems like anaerobic (irrigated rice), temporarily rainfed (lowland rice or floating rice) and aerobic (upland rice) conditions. Conventionally, rice is transplanted that requires standing water, the availability of which will be a major concern in near future. Aerobic cultivation has emerged as an alternative wherein the crop is raised direct seeded and need based irrigation is provided with proper management practices^1^. This kind of shift in rice cultivation is needed to cope with the water scarcity as well as to maintain the ground water table. Rice improvement strategies thus need to be focused on development of input use efficient genotypes. Only a few cultivars are known adapt to minimal water requirement and yield sustainably under aerobic conditions.

Understanding the molecular mechanisms and the genomic regions associated with the adaptation is necessary to design rice improvement strategies especially for water availability and uptake. Aerobic rice varieties have been developed by crossing the drought tolerant cultivars with high yielding cultivars and certain QTLs have also been mapped for yield and root-related traits under water deficit conditions^2,3^. Information on the genes that are responsible for various metabolic activities that confer the aerobic adaptation is sparsely available. Nevertheless, notable differences have been observed in several traits like root characters, root establishment, panicle initiation and flowering time etc. under both the systems of cultivation.

With the advent of next-generation sequencing, RNA-seq has become very useful to analyze the gene expression and molecular mechanisms underlying various traits. RNA-seq has been widely employed in rice to understand its response to drought, salinity, developmental stages, biotic stresses etc^4^. Apart from the differential transcript expression, RNA-seq also provides information on the variant identification and alternative splicing. Differentially expressed transcripts can be mapped to the reported QTLs for specific traits to identify the best QTLs, which can be used in the molecular breeding programs. Among the several traits root system architecture is known to play a critical role in adaptation and hence we have focused on the root system and the developing shoot for unraveling the molecular mechanisms associated with the aerobic adaptation.

## Results

### RNA-seq and key transcripts under aerobic/anaerobic conditions

RNA-seq of shoot and root tissues emanated into 3.12 billion raw reads (from BPT 5204 and CR Dhan 202) under aerobic and anaerobic conditions. Approximately, 37 million clean reads were generated from each shoot and root tissue sample. A total of 1.65 billion (88.28%) high quality clean reads of shoot and root tissue samples were mapped to the reference genome of *O*. *sativa* (*Nipponbare, japonica*) (Table 1). The alignment resulted into 1.38 billion (83.59%) of high quality reads mapping to the reference genome.

**Table 1:** Overview of mapping status/Summary statistics of RNA-seq.

Sample names	Raw reads	Total reads	Total mapped reads	Multiple mapped reads	Uniquely mapped reads
**Shoot**					
BAS	54286874	47112660	39401511 (83.63%)	1226119 (2.60%)	38175392 (81.03%)
CAS	39166536	35138424	29487654 (83.92%)	778878 (2.22%)	28708776 (81.70%)
BFS	42887614	38145326	31922697 (83.69%)	745441 (1.95%)	31177256 (81.73%)
CFS	50688434	44731418	37228814 (83.23%)	800911 (1.79%)	36427903 (81.44%)
**Root**					
BAR	34912250	32007740	27678083 (86.47%)	4324087 (13.50%)	23353996 (72.96%)
CAR	38186938	35151990	30221882 (85.97%)	1577386 (4.48%)	28644496 (81.48%)
BFR	29217686	27613644	23581800 (85.39%)	577246 (2.09%)	23004554 (83.30%)
CFR	35698678	31962918	27698548 (86.65%)	1829328 (5.72%)	25869220 (80.93%)

**Figure 1:**
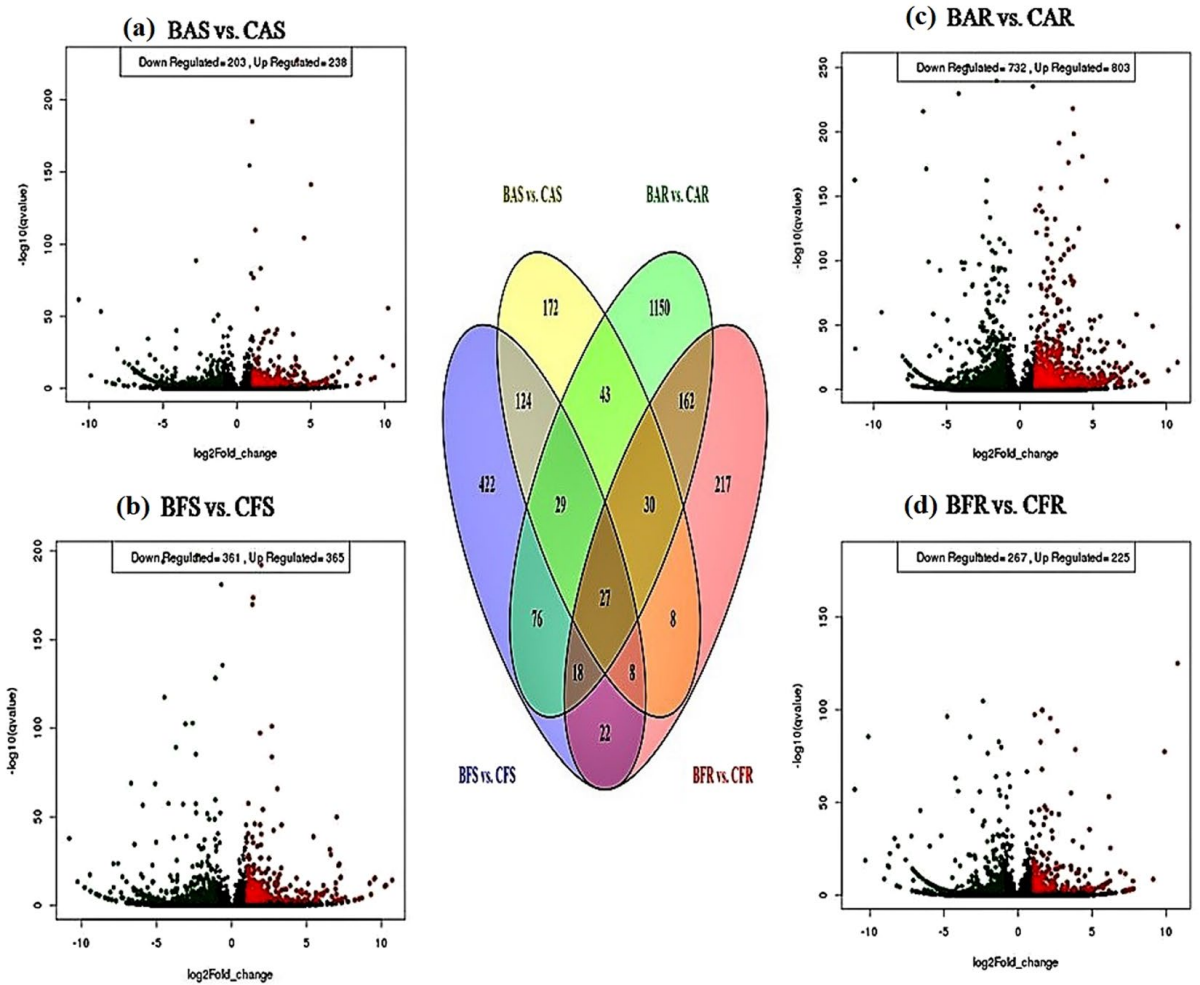
Venn diagram of the differentially expressed transcripts (DETs) in shoot (BAS vs. CAS, BFS vs. CFS) and root (BAR vs. CAR, BFR vs. CFR) of cultivars BPT 5204 and CR Dhan 202 under aerobic and anaerobic conditions. Volcano plots for differentially expressed transcripts (DETs) represents the logarithm of fold change (log_2_) on x-axis and log10 of the q-value of each transcripts on y-axis. In shoot (**a**) BAS vs. CAS (203 Down-regulated, 238 Up-regulated), (**b**) BFS vs. CFS (361 Down-regulated, 365 Up-regulated) and in root (**c**) BAR vs. CAR (732 Down-regulated, 803 Up-regulated), (**d**) BFR vs. CFR (267 Down-regulated, 225 Up-regulated).

Higher number of differentially expressed transcripts (DETs) were observed in root tissue under aerobic condition (BPT 5204 Aerobic Root, BAR vs. CR Dhan 202 Aerobic Root, CAR) than the anaerobic condition (BPT 5204 Anaerobic Root, BFR vs. CR Dhan 202 Anaerobic Root, CFR). Among these, higher number DETs were observed in aerobic adapted cultivar, AAC (CR Dhan 202) than non-adapted cultivar, NAC (BPT 5204). While in shoot tissue, lower number DETs were observed under aerobic condition (BPT 5204 Aerobic Shoot, BAS vs. CAS, CR Dhan 202 Aerobic Shoot) than the anaerobic condition (BPT 5204 Anaerobic Shoot, BFS vs. CR Dhan 202 Anaerobic Shoot, CFS), but in AAC higher number DETs were found than the NAC. A total of 188 transcripts in shoot and 237 in root tissues were found to be common between AAC and NAC under both the conditions (Fig. 1; Supplementary Table S1).

Gene Ontology (GO) enrichment was performed for DETs in shoot and root tissues to gain insights into their involvement in various functional annotations under aerobic and anaerobic conditions. The DETs were categorized into three major groups *viz*. biological process (BP), molecular function (MF), and cellular component (CC) gene ontologies (GO) and were assigned to GO numbers. A total of 274 in shoot (BAS vs. CAS) and 394 in root (BAR vs. CAR) GO-enriched DETs were identified in aerobic condition. Among those, majority (81.38%, 58.12%) were categorized into biological process followed by molecular function (16.05%, 36.29%) and cellular components (2.55%, 5.58%) in shoot and root tissues, respectively. Among the biological function, transcripts responding to water deprivation (GO: 0009414), cold (GO: 0009409) and response to stress (GO: 0006950), were significantly enriched. Transcripts related to nucleic acid binding transcription factor (GO: 0001071) and oxido reductase activity (GO: 0016491) were significant in molecular function category. Transcripts related to extracellular region (GO: 0005576) and cytoplasmic membrane-bound vesicle (GO: 0016023) were enriched under cellular component in both root and shoot tissues (Supplementary Fig. S1).

To know possible metabolic pathways involved in aerobic adaptation metabolic pathway enrichment was carried out through KEGG (Kyoto Encyclopedia of Genes and Genomes) database. In aerobic condition, metabolic overview pathway (osa01100) and biosynthesis of secondary metabolites (osa01110) were the most significantly enriched pathways in the root tissue. Fatty acid degradation (osa00071), glyoxylate and dicarboxylate metabolism (osa00630) were the most significant pathways represented in the shoot tissue. In addition, several other metabolic pathways such as glycolysis/gluconeogenesis, biosynthesis of amino acids like phenylalanine, tyrosine and tryptophan and phenylpropanoid biosynthesis were also significantly enriched in shoot (BAS vs. CAS) and root (BAR vs. CAR) under aerobic condition (Supplementary Table S2).

In addition to this, metabolic pathway analysis of DETs through MapMan also represented similar pathways *viz*. metabolic overview, biosynthesis of secondary metabolites (phenylpropanoids, flavonoids, terpenes, isopentenyl amino acids and nucleotides), photosynthesis, glycolysis, sucrose/starch metabolism, N-metabolism (amino acid and nucleotides, raffinose) in the shoot and root tissues under aerobic condition (Supplementary Fig. S2).

To get a better insight on the molecular mechanism of aerobic adaptation, we have functionally classified the transcripts differentially expressed under aerobic and anaerobic conditions into transcription factors (TFs), transporters and root trait related genes.

Nine TFs were exclusively expressed in the shoot tissue (BAS vs. CAS) under aerobic condition compared to anaerobic condition (Supplementary Fig. S3a). Among the exclusively expressed TFs, seven were highly up-regulated in AAC compared to NAC. Among these, five TFs *viz*. MADS4, MADS5, MADS6, MADS7 and putative WRKY transcription factor 6 were uniquely up-regulated in AAC. In root tissue, 46 TFs were exclusively expressed under aerobic condition compared to anaerobic condition (Supplementary Fig. S3b). Among the exclusively expressed TFs, twenty-one were highly up-regulated in AAC. Interestingly, among these, five TFs *viz*. MADS15, MADS26, Dehydration-responsive element-binding (DREB) family protein, DREB1F and DREB1C were uniquely up-regulated in AAC. Upon validation of expression through qRT-PCR, one of the uniquely expressed TFs i.e. DREB1F, was found to exhibit 1.51 fold higher expression in AAC under aerobic condition, which indicates the importance of TFs in aerobic adaptation.

Transcripts involved in ion transport at the early stages of panicle development in both root and shoot under aerobic condition were identified. In shoot tissue, only one monosaccharide transporter (Os07G0559700) was up-regulated in AAC compared to NAC (Supplementary Fig. S4a) under aerobic condition. Nineteen transporters were exclusively expressed in the root tissue. Among those, eleven transporters were highly up-regulated and eight transporters *viz*. bidirectional sugar transporters (SWEET 16, 3A and 4), cation (HKT8), inorganic phosphate (PHT1; 6), phosphate (PHO1;2), MDR-like ABC and vacuolar iron transporter homolog 2 transporter were uniquely up-regulated in AAC (Supplementary Fig. S4b). Further, validation of expression through qRT-PCR of two uniquely expressed transporters *viz*. inorganic phosphate (PHT1;6) and phosphate (PHO1;2) revealed 4.42 and 2.55 fold higher expression in AAC than NAC, respectively under aerobic condition. Transporters were found to be highly responsive in the root tissue compared to that in the shoot at panicle initiation stage under aerobic condition.

### Roots: The hidden half

Twenty-three root trait related genes were exclusively expressed under aerobic condition compared to anaerobic condition in the root tissue. Among those, twelve genes were up-regulated in the AAC than in the NAC, and seven genes were uniquely up-regulated. Three metallothionein (MT) genes *viz*. *OsMT2A* (Os01G0149800), *OsMT2B* (Os01G0974200) and *OsMT2C* (Os05G0111300) which are known to be involved in crown root formation were uniquely up-regulated in the AAC (Supplementary Table S3). Among these genes, *OsMT2A* (Os01G0149800) was further validated through the qRT-PCR which revealed higher expression in AAC (2.96 fold in root) than NAC. Two genes related to nutrient uptake including P uptake (*OsPHT1;6*), K and Na uptake (*OsHKT8*) which are known to have direct role in the roots were also uniquely up-regulated in NAC (Supplementary Table S3). Genes related to root hair development *viz*. *OsEXPB3* (Os10G0555900) and aquaporin gene *viz*. *OsPIP2;3* (Os04G0521100) were uniquely up-regulated in AAC compared to NAC (Supplementary Table S3).

### Role of Alternative splicing (AS) events

Alternative splicing (AS) is a key mechanism for the regulation of gene expression that increases transcriptional complexity and diversity. We investigated the influence of aerobic condition on alternative splicing events. Five types of AS events *viz*. skipped exon (SE), retained intron (RI), alternative 5′ splice site (A5′SS), alternative 3′ splice site (A3′SS) and mutual exclusive exons (MXE) were identified in shoot and root under aerobic and anaerobic conditions. Interestingly, higher number of AS events were observed in shoot and root tissues in both the cultivars in aerobic than in the anaerobic condition. Among the five AS events, retained intron (RI) type of AS events was the most prevalent in the both the tissues under the aerobic condition (Supplementary Table S4). However, novel AS isoforms were also found under both the conditions in shoot and root tissues. More AS events were observed in particular transcripts *viz*. tetratrico peptide repeat (TPR) domain containing protein (Os03G0308800) and GOLDEN2-LIKE1 (GLK1) transcription factor (Os06G0348800) in the shoot tissue. Similarly, a transcript (Os01G0363500) related to the gene coding for PAP fibrillin domain containing protein showed four different types of AS events such as RI, A5′SS, A3′SS and SE in root under aerobic condition. Upon validation through qRT-PCR, we observed the higher expression of TPR and GLK1 (1.12 and 1.63 fold respectively) in the shoot of AAC than NAC.

### Transcripts underlying known QTLs

To identify the probable genes underlying the reported QTLs for water deficit conditions, *in-silico* mapping of the uniquely up-regulated transcripts of AAC was carried out using QTARO database (Table 2). MADS4 (Os05G0423400) was mapped with two root trait related QTLs *viz*. qrfw1b and qbrt1d, reported earlier. Similarly, HKT8 (Os01G0307500), MDR-like ABC transporter (Os01G0723800) and vacuolar iron transporter homolog 2 (Os04G0538400) were co-localized with three root trait QTLs *viz*. qrfw1b, qbrt1d and qrfw4a, respectively. Another root trait related QTL qRDW5-1 co-localized with MPK7 (mitogen-activated protein kinase 7; Os05G0566400) on chromosome-5.

**Table 2:** Uniquely expressed transcripts in shoot and root of aerobic adapted cultivar CR Dhan 202 underlying QTLs for root related traits and water deficit tolerance.

DETs	Description	Start (bp)	End (bp)	QTLs	Start (bp)	End (bp)
**(a) In shoot (CAS)**						
Os05G0423400	MADS box transcription factor 4	20327992	20334833	brt1d	14045859	42715593
**(b) In root (CAR)**						
Os01G0307500	Cation transporter HKT8	11458956	11463442	rfw1b	7476658	35715948
Os01G0723800	MDR like ABC transporter	30189384	30195340	rfw1b	7476658	35715948
Os01G0723800	MDR like ABC transporter	30189384	30195340	brt1d	14045859	42715593
Os04G0538400	Vacuolar iron transporter homolog 2	26931464	26932639	rfw4a	25626203	33083265
Os05G0566400	Mitogen-activated protein kinase 7	28188894	28194022	qRDW5-1	27056983	28855659

brt- basal root thickness; rfw- root fresh weight; rdw- root dry weight.

### Validation of differentially expressed transcripts

**Figure 2:**
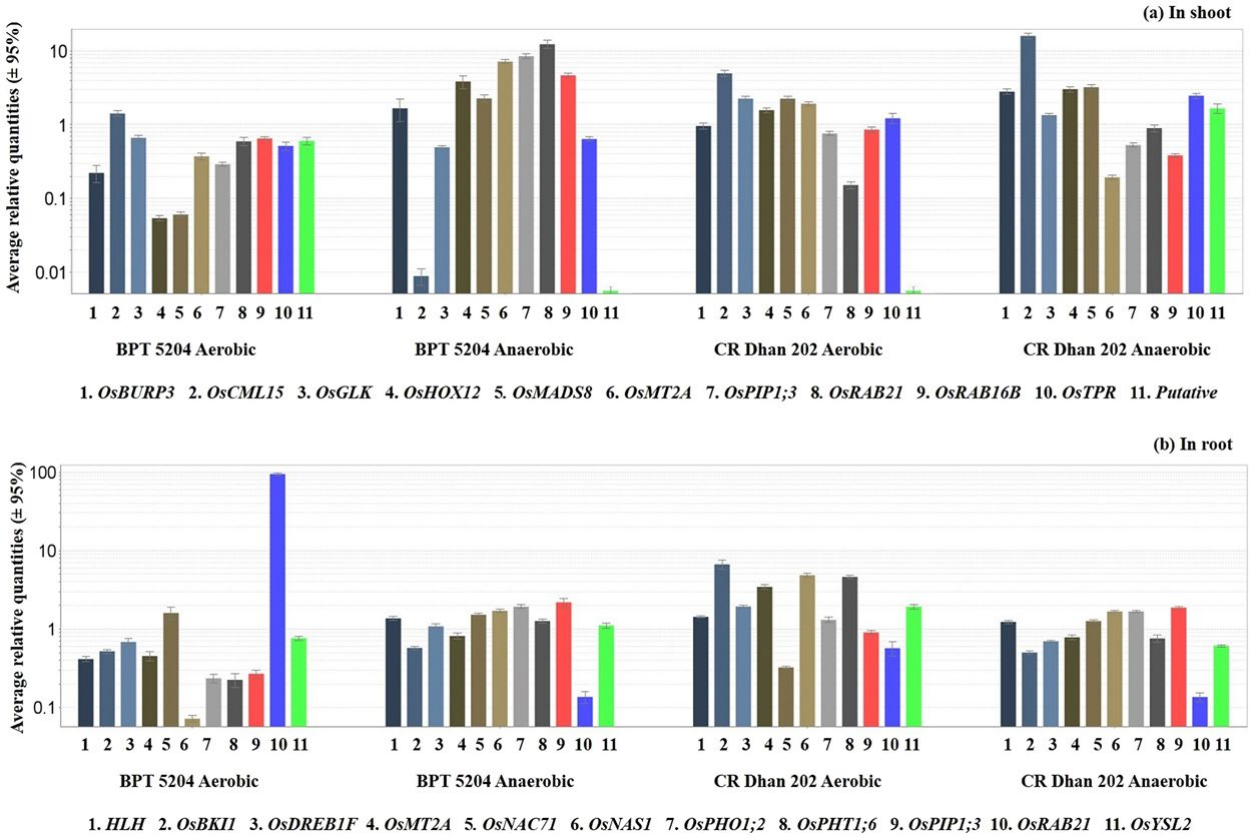
Relative expressions of representative transcripts in shoot (BAS vs. CAS, BFS vs. CFS) and root (BAR vs. CAR, BFR vs. CFR) under aerobic and anaerobic conditions. Name of primers (**a**) In shoot *viz*. 1. *OsBURP3*, 2. *OsCML15*, 3. *OsGLK1*, 4. *OsHOX12*, 5. *OsMADS8*, 6. *OsMT2A*, 7. *OsPIP1;3*, 8. *OsRAB21*, 9. *OsRAB16B*, 10. *OsTPR*, 11. *Putative* and (**b**) In root *viz*. 1. *HLH*, 2. *OsBKI1*, 3. *OsDREB1F*, 4. *OsMT2A*, 5. *OsNAC71*, 6. *OsNAS1*, 7. *OsPHO1;2*, 8. *OsPHT1;6*, 9. *OsPIP1;3*, 10. *OsRAB21*, 11. *OsYSL2*. Normalized with set of three reference genes *viz*. *Exp1* (Expressed protein), *TPH* (Tumor protein homolog) and *Memp* (Membrane protein). Error bar indicates the standard deviation of three technical replicates (n = 3).

The DETs obtained from RNA-seq data were validated by quantitative real time PCR (qRT-PCR) analysis. The expression of eleven functionally significant transcripts in shoot and root was validated through qRT-PCR analysis (Fig. 2). We observed similar expression patterns of transcripts (up and down regulation) in shoot (correlation coefficient of 0.966) and root (correlation coefficient of 0.994) between qRT-PCR and RNA-seq data under aerobic condition. The expression pattern of qRT-PCR correlated very well with that of RNA-seq data (Supplementary Fig. S5).

### Mechanism of aerobic adaptation

**Figure 3:**
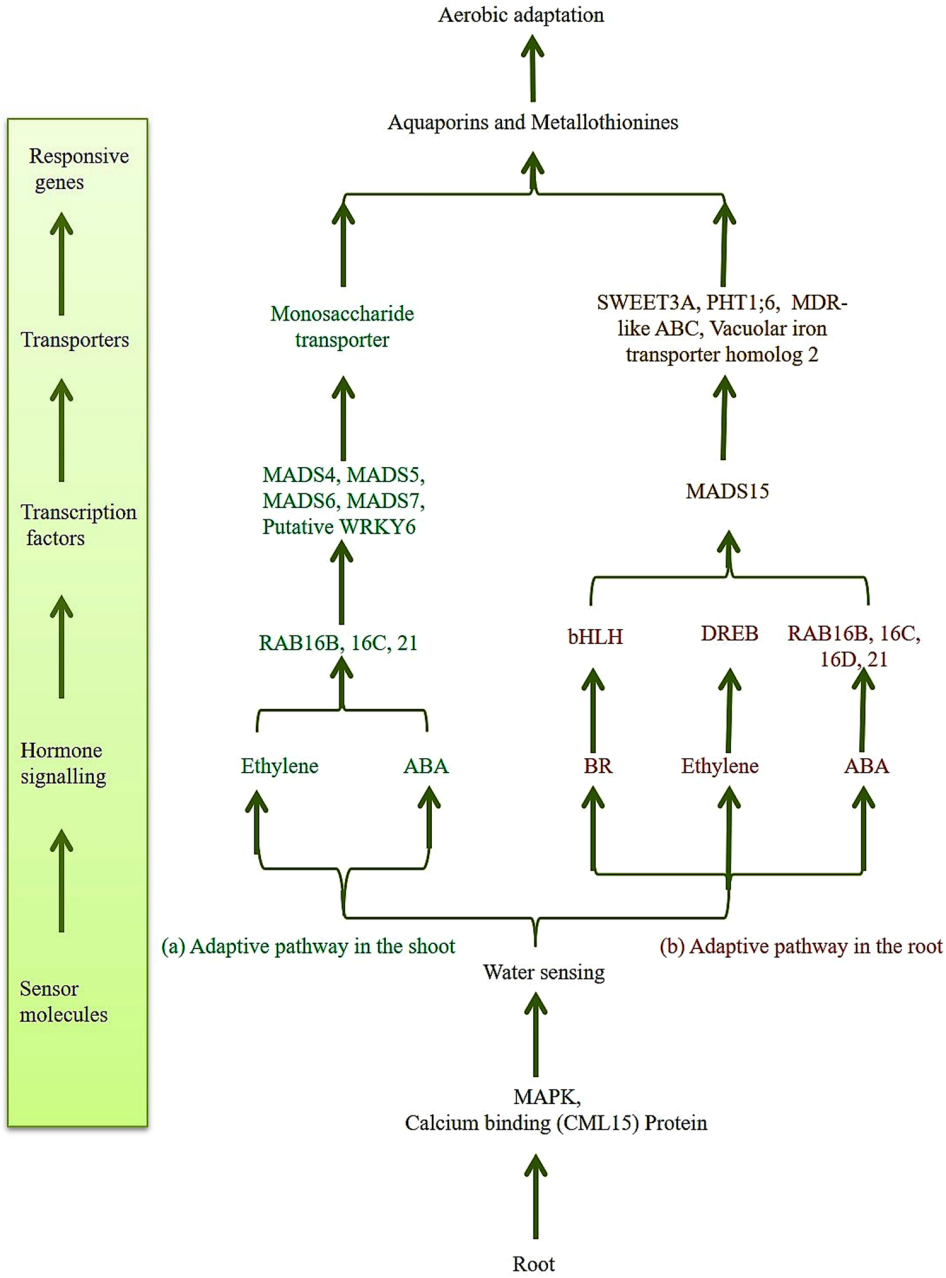
Proposed pathway of aerobic adaptation in cultivar CR Dhan 202. Pathway represents the uniquely expressed different class of key genes which includes TFs, transporters and root traits related genes in shoot and root tissue in aerobic condition of cultivar CR Dhan 202.

We have identified the key candidate genes and revealed the aerobic adaptation mechanism in the AAC (Fig. 3). Based on these observations a possible mechanism of aerobic adaptation is proposed. In aerobic condition, root tissue sensing the water limiting condition initiates the expression of transcripts related to sensor molecules like mitogen activated protein kinase (MAPK) and calcium binding proteins. The plant sends the molecular signals from the roots to the shoots by increasing the expression of transcripts responsible for hormonal signaling including ethylene, abscisic acid (ABA) and brassinosteroid (BR), which leads to the expression of abscisic acid responsive transcripts *viz*. RAB21, RAB16B and RAB16C. These transcripts might be responsible for regulating the expression of MADS family TFs *viz*. MADS4, MADS5, MADS6, MADS7 and putative WRKY transcription factor 6. Further, it can be extended that these key TFs are responsible for regulating the expression of genes such as monosaccharide transporter and the root trait-related aquaporins and metallothionines contributing to the aerobic adaption (Fig. 3a).

Similarly, in the root tissue hormonal signaling by ethylene, ABA and brassinosteroid operates that leads to the expression of response to abscisic acid (RAB) and dehydration-responsive element-binding (DREB) transcripts. Further, combined expression of RAB and DREB transcripts might regulate the expression of key MADS15 transcription factor. The MADS15 plays a role in regulating the expression of genes that include key transporters (SWEET3A, PHT1;6, MDR-like ABC and vacuolar iron transporter homolog 2) and those related to the root traits (aquaporins and metallothionines) leading to the aerobic adaptation (Fig. 3b).

The aerobic adaptation mechanism partially mimics that of drought tolerance or it shares the common pathway to manifest the desired response in the AAC. Thus, the mechanism of aerobic adaptation can be conferred by the combined expression of key candidate genes related to the hormonal signaling, transcription factors, transporters and root trait related genes in AAC.

## Discussion

RNA-seq based transcriptome profiling of two rice cultivars, AAC (CR Dhan 202) and NAC (BPT 5204) in shoot and root tissues was performed at panicle initiation (PI) stage under aerobic and anaerobic conditions. Panicle initiation (PI) stage is considered the crucial stage, which is affected by various abiotic stresses, especially water deficit condition leading to the yield loss^5^. RNA-seq is an attempt to understand cultivar specific gene expression and identify the key genes, which reveal the molecular mechanism of aerobic adaptation. A higher number of differentially expressed transcripts (DETs) have been observed in the root than in the shoot under aerobic condition. It implies that the roots play an important role in adapting to the aerobic condition. From the GO enrichment results, it can be inferred that most of the terms appeared under aerobic condition corroborated with the enriched terms reported in various RNA-seq studies under oxidative stress, drought tolerance and cold stress etc.^4,6–10^. It can be envisaged that a cross-talk mechanism under aerobic condition might exist among the pathways involved in the responses to different abiotic stresses^11,12^. It was also observed that the major pool of transcripts expressed under aerobic conditions matches with the reported stress related transcripts^4,6–10^. Thus, it was quite evident that there exists a role of transcriptional reprogramming for aerobic adaptation in rice. The metabolic overview pathway and the biosynthesis of secondary metabolites might play a crucial role in imparting the adaptation to the aerobic condition.

Stress-responsive TFs have been considered key regulators in plants under various abiotic stress conditions^13,14^. The uniquely expressed five TFs in shoot *viz*. MADS4, MADS5, MADS6, MADS7 and putative WRKY transcription factor 6 and one TF in root *viz*. MADS15 of AAC was not reported earlier in any stress conditions. However, their expression was reported in the panicle tissue^15^. It has been reported that MADS6 acts as an upstream regulator that activates the expression of MADS7 and MADS8 during early flower development^16^. Genome wide association study (GWAS) for grain yield under water deficit also indicated that the association of single nucleotide polymorphic markers (SNP) in the genes coding for AP2 and WRKYs^17^. Uniquely higher expression of MADS26 observed in the present study corroborated with the earlier report of drought stress^18^. Other than MADS26, uniquely expressed TFs in root i.e. DREB1F and DREB1C have been previously reported under cold, dehydration, drought, salinity and low temperature stress tolerance in rice^19,20^, which indicated that certain drought tolerance characteristics may also contribute to the aerobic adaptation^14^. Our results suggest that the possible involvement of the members of MADS-TF family along with WRKY transcription factor 6 imparts aerobic adaptation in AAC. The key TFs involved in the aerobic adaptation need further attention.

Transporter genes are known to play a vital role in ion transport and homeostasis during several abiotic stresses. Among the eight uniquely expressed transporters in the AAC, two transporters *viz*. SWEET16 and SWEET4 are reported to be involved in cold and drought stresses in *Arabidopsis*^21,22^. Interestingly the involvement of SWEET3A was not reported earlier in any of the stress responses. Two nutrient uptake transporters *viz*. HKT8 and PHO1;2 were reported in salt stress and phosphorous deficiency in rice^23,24^. However, involvement of PHT1;6, MDR-like ABC and vacuolar iron transporter homolog 2 were not reported under any stress responses indicating their unique role in aerobic adaptation and PHT1;6 was further validated in our qRT-PCR assay. In general, the aerobic condition leads to iron and zinc deficiencies and the adapted cultivars can overcome the deficiency by expressing the required transporters for efficient uptake^25^. This observation matched with our earlier studies where certain nutrient uptake genes were highly expressed in AAC than in NAC under aerobic condition at panicle initiation stage^26^.

Crown root forming gene *viz*. metallothionein-like protein 2B (Os01G0974200) and root hair development gene *viz*. *OsEXPB3* were not reported under any stress conditions, which implies that these genes help specifically in root growth and development under aerobic condition. This observation also matched with our earlier report on root morphology where the root length in AAC was found to be significantly higher than that of the NAC^27^. Other uniquely expressed root specific genes *viz*. *OsMT2A* and *OsMT2C* and aquaporin *OsPIP2;3* were reported to be expressed in heavy metal stress^28,29^ and drought stress^30^. The qRT-PCR assay also revealed the higher expression of *OsMT2A* in AAC but not in NAC.

Alternative splicing (AS) mechanism operates in higher eukaryotes during various developmental processes and in response to environmental stimuli^31,32^. In our study, retained intron (RI) was the most prevalent AS event under aerobic condition similar to that reported in various abiotic stresses (salt and heat)^31,33,34^. The higher frequency of AS events in shoot and root tissues under aerobic condition has been corroborated with its role in regulation under desiccation, salinity and drought stress tolerance in rice^34,35^. The AS has also been reported to operate in the abscisic acid (ABA) signaling as well as abiotic stress responses^36^. The prevalence of higher number of AS events in tetratrico peptide repeat (TPR) and GOLDEN2-LIKE1 (GLK1) genes was reported in cold stress^37^, hormone signaling^38^ and plant and chloroplast development^39,40^. Our qRT-PCR assay also revealed the higher expression of TPR and GLK1 in AAC but not in NAC. Thus, the presence of AS events might occur and alternative splice variants are generated as a result of aerobic adaptation.

Interestingly, uniquely expressed key TFs and transporters in AAC were mapped to some of the root related traits and drought stress tolerance QTLs^2,41–45^ such as qrfw4a, qrfw1b, qbrt1d and qRDW5-1. These QTLs might be operating in AAC and the mapped transcripts may serve as candidate genes for QTLs. These potential QTLs can be introgressed into NAC cultivars to generate high yielding AAC rice.

Based on our results, the proposed pathway of aerobic adaptation in AAC (CR Dhan 202) may operate by the initial signals emanating from the root, differential hormonal regulation that leads to the expression of specific transcription factors which induce the transporters and specific genes imparting adaptation to aerobic conditions.

In summary, our study provides a comprehensive overview of the transcriptome of two rice cultivars grown under aerobic and anaerobic conditions, which revealed the molecular mechanism underlying the adaptation to aerobic condition. Unique genes specific to the aerobic adaptation have been identified and some found to be located in water deficit related QTLs. Overall, the genes identified are useful resources to carry out future studies on genetic improvement of rice for aerobic conditions.

## Methods

### Plant material and treatments

Rice (*Oryza sativa* L.) genotypes CR Dhan 202 (AAC) and BPT 5204 (NAC) were used in this study. The seeds were sown in the polythene bags containing 15 kg soil by maintaining aerobic and anaerobic conditions in the greenhouse with 28/20 °C day/night temperature. Anaerobic condition was maintained by keeping two-five cm water above the soil for a period of 100–120 days, while aerobic condition was maintained by keeping the moisture at field capacity and maintaining the well drained soil all the time. Water in measured volume was given as and when required to maintain the above said conditions. The nutrients (NPK) equivalent to 15 kg soil was applied by following regular recommendations. At a panicle initiation stage (60–70 days after germination), shoot and root tissue samples were collected in three independent biological replicates and stored immediately in liquid nitrogen. The samples were abbreviated as BPT 5204 Aerobic Shoot (BAS), CR Dhan 202 Aerobic Shoot (CAS), BPT 5204 Anaerobic Shoot (BFS), CR Dhan 202 Anaerobic Shoot (CFS), BPT 5204 Anaerobic Root (BFR), BPT 5204 Aerobic Root (BAR), CR Dhan 202 Aerobic Root (CAR) and CR Dhan 202 Anaerobic Root (CFR).

### RNA isolation, cDNA library construction and Illumina sequencing

Total RNA was isolated from shoot and root tissue samples of the two cultivars using NucleoSpin RNA Plant kit (Macherey-Nagel, Duren, Germany) by following manufacturer’s protocol with slight modifications in three replications and pooled together. The quantity and quality of RNA samples were assessed by using ND1000 spectrophotometer (Thermo Scientific) and 1% (w/v) agarose gel. The RNA integrity number (RIN) and concentration were checked using an Agilent 2100 Bioanalyzer (Agilent Technologies, Inc., Santa Clara, CA, USA), wherein RIN values were >8.0 for all eight samples used for sequencing. Five μg of total RNA was used for cDNA library preparation and sequencing. The pair-end sequencing libraries were prepared using Illumina HiSeq2000 RNA Library Preparation Kit as per manufacturer’s protocol (Illumina®, San Diego, CA). The cDNA libraries were individually prepared from each sample by performing a series of procedures including poly(A) enrichment, RNA fragmentation, random hexamer primed cDNA synthesis, linker ligation, size selection and PCR amplification. The quality and quantification of cDNA libraries were performed by using the Qubit 2.0 Fluorometer (Thermo Scientific) and Agilent 2100 Bioanalyzer (Agilent Technologies, Singapore). The libraries were then sequenced using HiSeq Illumina 2500 sequencing platform (Illumina, San Diego, CA) at Nucleome Informatics Pvt. Ltd. Hyderabad (Supplementary Fig. S6).

### Pre-processing and reference mapping

The raw reads were filtered using NGSQC Toolkit^46^ using default parameters by removing low-quality bases (>Q30), adapter contaminated reads. The resulting high-quality clean reads were mapped to Nipponbare (IRGSP-1.0 pseudomolecule/MSU7) reference genome using TopHat (V 2.0.13) using default parameters^47^. The resulting alignment (in BAM file format) was used to generate transcript annotations (GTF format) with the Cufflinks (V 2.2.1) using default parameters^48,49^. Gene expression levels were estimated using FPKM values (Fragments Per Kilobase of transcript per Million fragments mapped) by the Cufflinks (V 2.2.1) software.

## Gene expression analysis

The HTSeq was used to compare transcripts expression level between different samples on the basis of FPKM from reference-guided mapping. The log_2_ fold changes of transcripts FPKM between samples were tested statistically to determine whether an individual transcripts expression was altered significantly or not. The transcripts with false discovery rate (FDR of 0.005 and p-value ≤ 0.001) and log_2_ fold change ≥1.5 (up-regulated genes) and ≤(−1.5) (down-regulated genes) were considered as significantly differentially expressed transcripts (DETs) under aerobic and anaerobic conditions. The DETs were functionally classified and represented as heat map using Multi-experiment Viewer (MeV) v4.9.0^50^. The number of DETs among and within conditions was plotted as Venn diagram using Venny 2.1.0 (http://bioinfogp.cnb.csic.es/tools/venny/)^51^.

### Gene ontology (GO) enrichment and pathway analysis of transcripts

Gene ontology (GO) enrichment analysis was performed for the functional categorization of various differentially expressed transcripts using GOseq analysis tool^52^, which is based on Wallenius non-central hyper-geometric distribution. Pathway analysis of differentially expressed transcripts involved in specific pathways was carried out using KEGG (Kyoto Encyclopedia of Genes and Genomes) database^53^ and were plotted using MapMan (version 3.5.1; http://mapman.gabipd.org/web/guest) with P-value cut-off of ≤0.05^54^.

### Alternative splice variant identification

Alternative splicing (AS) events were determined using rMATS (replicate multivariate analysis of transcript splicing) in the differentially expressed transcripts with a stringent threshold of (|IncLevelDifference| ≥ 0.1 and P ≤ 0.05)^55^. The alternative splicing events were classified as skipped exons (SE), alternative 5′ splice site (A5SS), alternative 3′ splice site (A3SS), mutually exclusive exons (MXE) and retained intron (RI).

### QTL mapping

The DETs were mapped to the QTLs reported in the QTARO database (http://qtaro.abr.affrc.go.jp/qtab/table) using Bedtools (http://bedtools.readthedocs.io/en/latest/) which compare large sets of genomic feature by intersecting two interval files.

### Validation of transcripts

An aliquot of total RNA was used for synthesizing cDNA using PrimeScript^TM^ first strand cDNA synthesis kit (Takara, Japan) following the manufacturer instructions. The representative differentially expressed transcripts on the basis of function were selected and primers were designed using high throughput qRT-PCR tool, QuantPrime (http://quantprime.mpimp-golm.mpg.de/) available online keeping the default parameters. The details of primers for shoot and root are summarized in Supplementary Table S5.

The qRT-PCR reactions were performed on a Light Cycler 96 real-time PCR system (Roche, USA) using the SYBR Premix ExTaq^TM^ II (Takara, Japan). Each reaction was performed in triplicate containing 5 µl SYBR Green Master, 0.8 µl template cDNA, 0.4 µl each of the primers (10 µM), and 3.4 µl RNase-free water with a total volume of 10 µl. The qRT-PCR profile for differentially expressed transcripts was as follows such as 95 °C for 2 min followed by 40 cycles of 95 °C for 5 s, 60 °C for 30 s with fluorescent signal recording and 72 °C for 30 s. The melting curve was obtained using a high resolution melting profile performed after the last PCR cycle: 95 °C for 15 s followed by a constant increase in the temperature between 65 °C for 15 s and 95 °C for 1 s. A set of three reference genes *viz*. *Exp1* (Expressed protein), *TPH* (Tumor protein homolog) and *Memp* (Membrane protein) was used as internal control genes in root and shoot tissues at panicle initiation stage under aerobic and anaerobic conditions as reported in our previous study^26^. The relative expression levels of transcripts in the shoot and root samples were recorded using qBase plus software^56,57^. The gene expression patterns of transcripts obtained from both qRT-PCR and RNA-seq analysis were plotted in Microsoft excel as a correlation graph.

## Supplementary information

Supplementary Information

Dataset 1

## Data Availability

RNA-seq data generated in the study have been deposited in the National Center for Biotechnology Information (NCBI) under the accession codes of Bio Project ID: PRJNA414373 and SRA submission ID: SRP120096.

## References

[1] Pathak H (2011). Direct-seeded rice: Potential, performance and problems - A review. Cur. Adv. Agri. Sci..

[2] Sandhu N, Jain S, Kumar A, Mehla BS, Jain R (2013). Genetic variation, linkage mapping of QTL and correlation studies for yield, root, and agronomic traits for aerobic adaptation. BMC Genetics..

[3] Sandhu N (2015). Traits and QTLs for development of dry direct-seeded rainfed rice varieties. J. Exp. Bot..

[4] Shankar R, Bhattacharjee A, Jain M (2016). Transcriptome analysis in different rice cultivars provides novel insights into desiccation and salinity stress responses. Sci. Rep..

[5] Wei H (2017). Comparative analysis of expression profiles of panicle development among tolerant and sensitive rice in response to drought stress. Front. Plant Sci..

[6] Guo H (2017). Transcriptome analysis of neo-tetraploid rice reveals specific differential gene expressions associated with fertility and heterosis. Sci. Rep..

[7] Gao Y, Xu H, Shen Y, Wang J (2013). Transcriptomic analysis of rice (Oryza sativa) endosperm using the RNA-Seq technique. Plant Mol. Biol..

[8] Baldoni E, Bagnaresi P, Locatelli F, Mattana M, Genga A (2016). Comparative leaf and root transcriptomic analysis of two rice japonica cultivars reveals major differences in the root early response to osmotic stress. Rice.

[9] Huang A, Sang Y, Sun W, Fu Y, Yang Z (2016). Transcriptomic analysis of responses to imbalanced Carbon: Nitrogen availabilities in rice seedlings. Plos One.

[10] Zhang ZF, Li YY, Xiao BZ (2016). Comparative transcriptome analysis highlights the crucial roles of photosynthetic system in drought stress adaptation in upland rice. Sci. Rep..

[11] Qin F, Shinozaki K, Yamaguchi-shinozaki K (2011). Achievements and challenges in understanding plant abiotic stress responses and tolerance. Plant Cell Physiol.

[12] Nakashima K, Yamaguchi-shinozaki K, Shinozaki K (2014). The transcriptional regulatory network in the drought response and its crosstalk in abiotic stress responses including drought, cold, and heat. Front Plant Sci..

[13] Nakashima K, Ito Y, Yamaguchi-Shinozaki K (2009). Transcriptional regulatory networks in response to abiotic stresses in Arabidopsis and grasses. Plant Physiol..

[14] Todaka D, Shinozaki K, Yamaguchi-Shinozaki K (2015). Recent advances in the dissection of drought-stress regulatory networks and strategies for development of drought-tolerant transgenic rice plants. Front Plant Sci.

[15] Arora R (2007). MADS-box gene family in rice: genome-wide identification, organization and expression profiling during reproductive development and stress. BMC Genomics.

[16] Li H (2011). Rice MADS6 interacts with the floral homeotic genes SUPERWOMAN1, MADS3, MADS58, MADS13, and DROOPING LEAF in specifying floral organ identities and meristem fate. Plant Cell..

[17] Pantaliao GF (2016). Genome wide association study (GWAS) for grain yield in rice cultivated under water deficit. Genetica.

[18] Khong GN (2015). OsMADS26 Negatively regulates resistance to pathogens and drought tolerance in rice. Plant Physiol.

[19] Dubouzet JG (2003). OsDREB genes in rice, Oryza sativa L., encode transcription activators that function in drought, high salt and cold responsive gene expression. Plant J..

[20] Wang Q (2008). Overexpression of a rice OsDREB1F gene increases salt, drought, and low temperature tolerance in both Arabidopsis and rice. Plant Mol. Biol..

[21] Chardon F (2013). Leaf fructose content is controlled by the vacuolar transporter SWEET17 in Arabidopsis. Curr Biol..

[22] Liu X, Zhang Y, Yang C, Tian Z, Li J (2016). AtSWEET4, a hexose facilitator, mediates sugar transport to axial sinks and affects plant development. Sci Rep.

[23] Ren ZH (2005). A rice quantitative trait locus for salt tolerance encodes a sodium transporter. Nat. Genet..

[24] Secco D, Baumann A, Poirier Y (2010). Characterization of the rice PHO1 gene family reveals a key role for OsPHO1;2 in phosphate homeostasis and the evolution of a distinct clade in dicotyledons. Plant Physiology..

[25] Shi R (2012). Responses of aerobic rice (Oryza sativa L.) to iron deficiency. J. Integr. Agric..

[26] Phule AS (2018). Genes encoding membrane proteins showed stable expression in rice under aerobic condition: novel set of reference genes for expression studies. 3 Biotech.

[27] Phule, A. S. *et al*. Studies on root anatomy, morphology and physiology of rice grown under aerobic and anaerobic conditions. *Physiol Mol Biol Plants*, **25**, 192 (2019). 10.1007/s12298-018-0599-z PMC6352520 30804642

[28] Kim YO, Kang H (2018). Comparative expression analysis of genes encoding metallothioneins in response to heavy metals and abiotic stresses in rice (Oryza sativa) and Arabidopsis thaliana. Biosci. Biotechnol. Biochem..

[29] Liu J (2015). Copper-induced hydrogen peroxide upregulation of a metallothionein gene, OsMT2c, from Oryza sativa L. confers copper tolerance in Arabidopsis thaliana. J. Hazard. Mater..

[30] Yamaguchi-Shinozaki K, Koizumi M, Urao S, Shinozaki K (1992). Molecular cloning and characterization of 9 cDNAs for genes that are responsive to desiccation in Arabidopsis thaliana: Sequence analysis of one cDNA clone that encodes a putative transmembrane channel protein. Plant Cell Physiol..

[31] Marquez Y, Brown JWS, Simpson C, Barta A, Kalyna M (2012). Transcriptome survey reveals increased complexity of the alternative splicing landscape in Arabidopsis. Genome Res..

[32] Zhang C, Yang H, Yang H (2015). Evolutionary character of alternative splicing in plants. Bioinform. Biol. Insights.

[33] Wang BB, Brendel V (2006). Genome-wide comparative analysis of alternative splicing in plants. Proc. Natl. Acad. Sci. USA.

[34] Chang CY, Lin WD, Tu SL (2014). Genome-wide analysis of heat-sensitive alternative splicing in physcomitrella patens. Plant Physiol..

[35] Wei H (2017). Alternative splicing complexity contributes to genetic improvement of drought resistance in the rice maintainer HuHan2B. Sci. Rep..

[36] Sugliani M, Brambilla V, Clerkx EJM, Koornneef M, Soppe WJJ (2010). The conserved splicing factor SUA controls alternative splicing of the developmental regulator *ABI3* in *Arabidopsis*. Plant Cell.

[37] Garapati P, Xue GP, Munné-Bosch S, Balazadeh S (2015). Transcription factor ATAF1 in Arabidopsis promotes senescence by direct regulation of key chloroplast maintenance and senescence transcriptional cascades. Plant Physiol.

[38] Sharma M, Pandey GK (2016). Expansion and function of repeat domain proteins during stress and development in plants. Front. Plant Sci..

[39] Waters MT (2009). GLK Transcription factors coordinate expression of the photosynthetic apparatus in Arabidopsis. Plant Cell Online.

[40] Tokumaru M (2017). Ubiquitin-proteasome dependent regulation of the GOLDEN2-LIKE 1 transcription factor in response to plastid signals. Plant Physiol.

[41] Venuprasad R (2009). Identification and characterization of large-effect quantitative trait loci for grain yield under lowland drought stress in rice using bulk-segregant analysis. Theor. Appl. Genet..

[42] Vikram P (2011). Reproductive-stage drought stress with a consistent effect in multiple elite genetic backgrounds. BMC Genet..

[43] Sandhu, N., Jain, S., Battan, K. R. & Jain, R. K. Aerobic rice genotypes displayed greater adaptation to water-limited cultivation and tolerance to polyethyleneglycol-6000 induced stress. **18** 33–43 (2012). 10.1007/s12298-011-0094-2 PMC3550527 23573038

[44] Sandhu N (2014). Identification and mapping of stable QTL with main and epistasis effect on rice grain yield under upland drought stress. BMC Genet..

[45] Li Z (2005). QTL mapping of root traits in a doubled haploid population from a cross between upland and lowland japonica rice in three environments. Theor Appl Genet.

[46] Patel, R. K. & Jain, M. NGS QC toolkit: A toolkit for quality control of next generation sequencing data. *PLoS One* **7** (2012). 10.1371/journal.pone.0030619 PMC3270013 22312429

[47] Trapnell C, Pachter L, Salzberg SL (2009). TopHat: Discovering splice junctions with RNA-Seq. Bioinformatics.

[48] Trapnell C (2010). Transcript assembly and quantification by RNA-Seq reveals unannotated transcripts and isoform switching during cell differentiation. Nat. Biotechnol..

[49] Roberts A, Pimentel H, Trapnell C, Pachter L (2011). Identification of novel transcripts in annotated genomes using RNA-seq. Bioinformatics.

[50] Saeed, A. I. *et al*. *TM4:* A free, open-source system for microarray data management and analysis. *BioTechniques* **34** (2003). 10.2144/03342mt01 12613259

[51] Oliveros, J. C. VENNY: an interactive tool for comparing lists with Venn Diagrams., http://bioinfogp.cnb.csic.es/tools/venny/index.html (2007).

[52] Young MD, Wakefield MJ, Smyth GK, Oshlack A (2010). Genome Biology Gene ontology analysis for RNA-seq: accounting for selection bias. Genome Biol.

[53] Kanehisa, M. & Goto, S. KEGG: Kyoto Encyclopedia of Genes and Genomes. **28**, 27–30 (2000). 10.1093/nar/28.1.27 PMC102409 10592173

[54] Thimm O (2004). MAPMAN: A user-driven tool to display genomics data sets onto diagrams of metabolic pathways and other biological processes. Plant J..

[55] Shen Y (2014). Global dissection of alternative splicing in paleopolyploid soybean. Plant Cell.

[56] Hellemans J, Mortier G, De Paepe A, Speleman F, Vandesompele J (2007). qBase relative quantification framework and software for management and automated analysis of real-time quantitative PCR data. Genome Bio.

[57] Livak KJ, Schmittgen TD (2001). Analysis of relative gene expression data using Real-Time Quantitative PCR and the 2−ΔΔCT method. Methods.

