# Supplementary material for: RNA-seq reveals the involvement of key genes for aerobic adaptation in rice
Source: Sci Rep. 2019 Mar 27;9:5235. doi: 10.1038/s41598-019-41703-2 (PMC6437204; doi:10.1038/s41598-019-41703-2)
Supplement: Supplementary file 1 — Supplementary Information [file 41598_2019_41703_MOESM1_ESM.pdf]

RNA-seq reveals the involvement of key genes for aerobic adaptation in rice

Amol S. Phule[ab], Kalyani M. Barbadikar[a], Sheshu Madhav Maganti[a], P. Seguttuvel[a], D. Subrahmanyam[a], M.B.B. Prasad Babu[a] and Polumetla A. Kumar[a*]

[a] Biotechnology Division, ICAR-Indian Institute of Rice Research, Hyderabad 30, India

[b] Institute of Biotechnology, Professor Jayashankar Telangana State Agricultural University, Hyderabad 30, India

*Correspondence: Polumetla Ananda Kumar

ICAR- Indian Institute of Rice Research

Rajendranagar, Hyderabad-500030

Telangana, India

E-mail: polumetla@hotmail.com

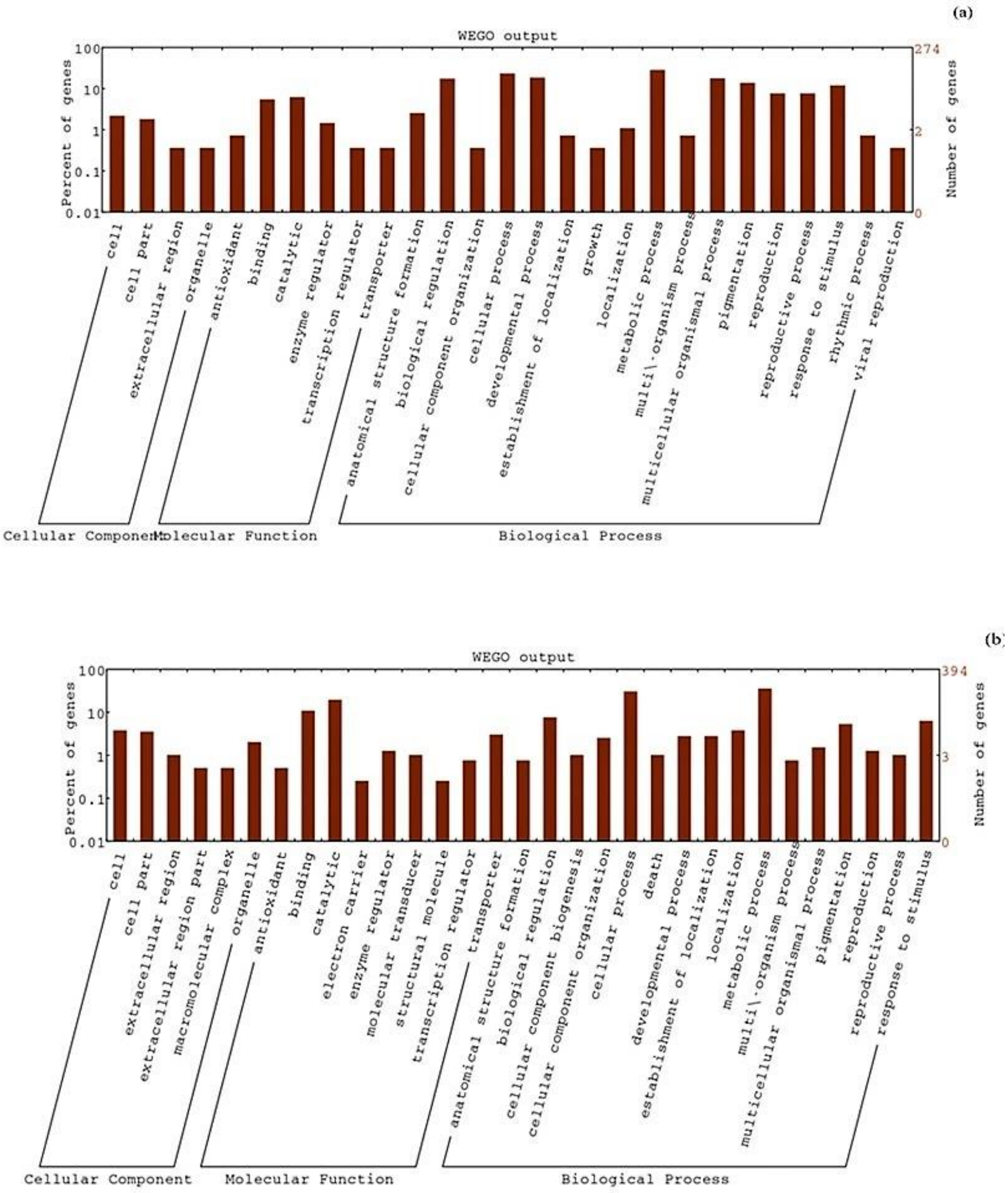

Fig. S1. GO enrichment analysis for DETs in shoot (BAS vs. CAS) and in root (BAR vs. CAR) under aerobic conditions. The categories of biological processes (BP) most significant followed by molecular function (MF) and cellular component GO terms in (a) shoot and (b) root are shown from top to bottom.

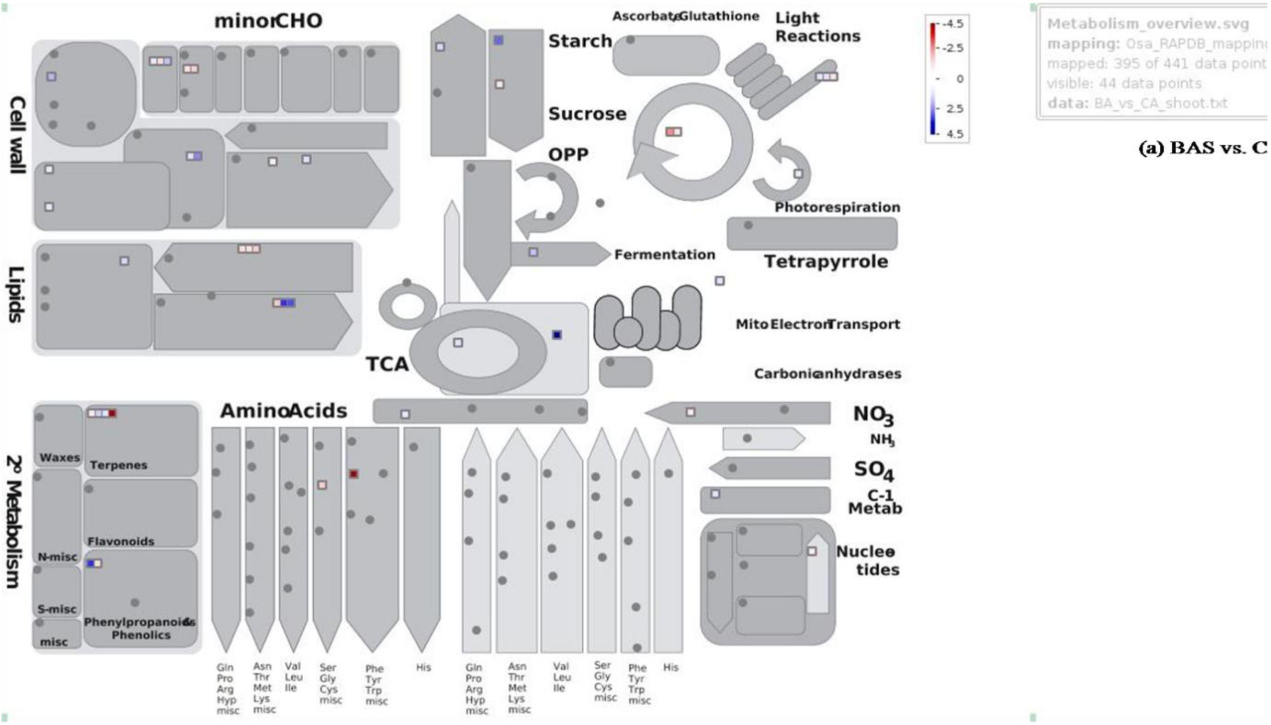

(a) BAS vs. C

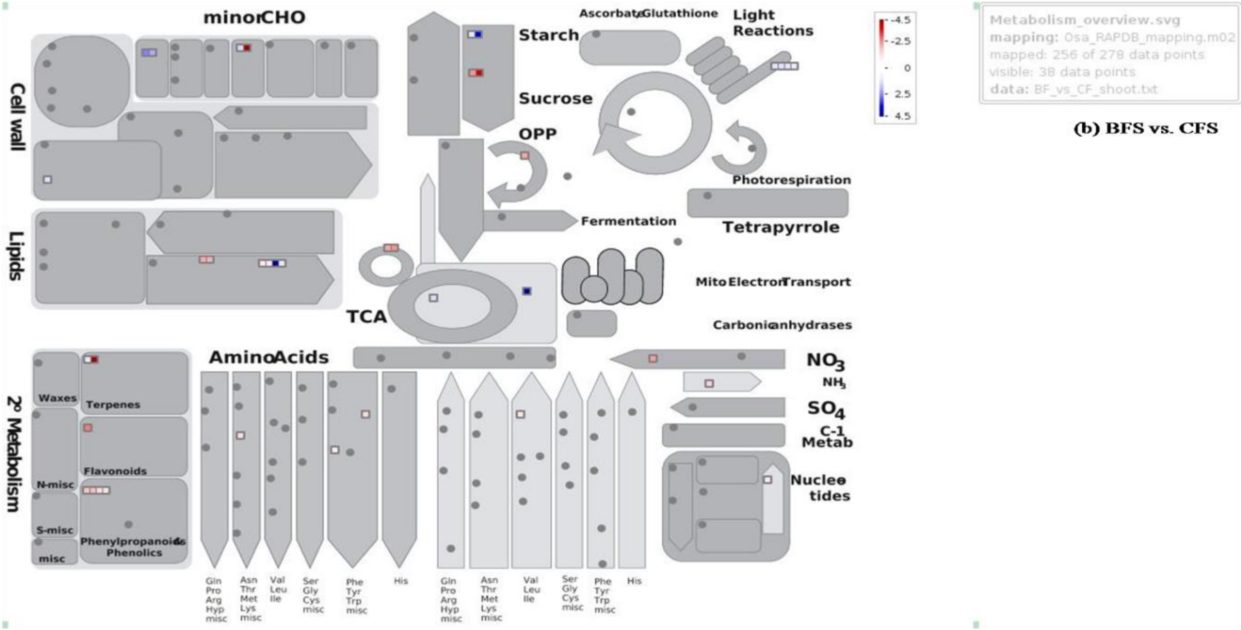

(b) BFS vs. CFS

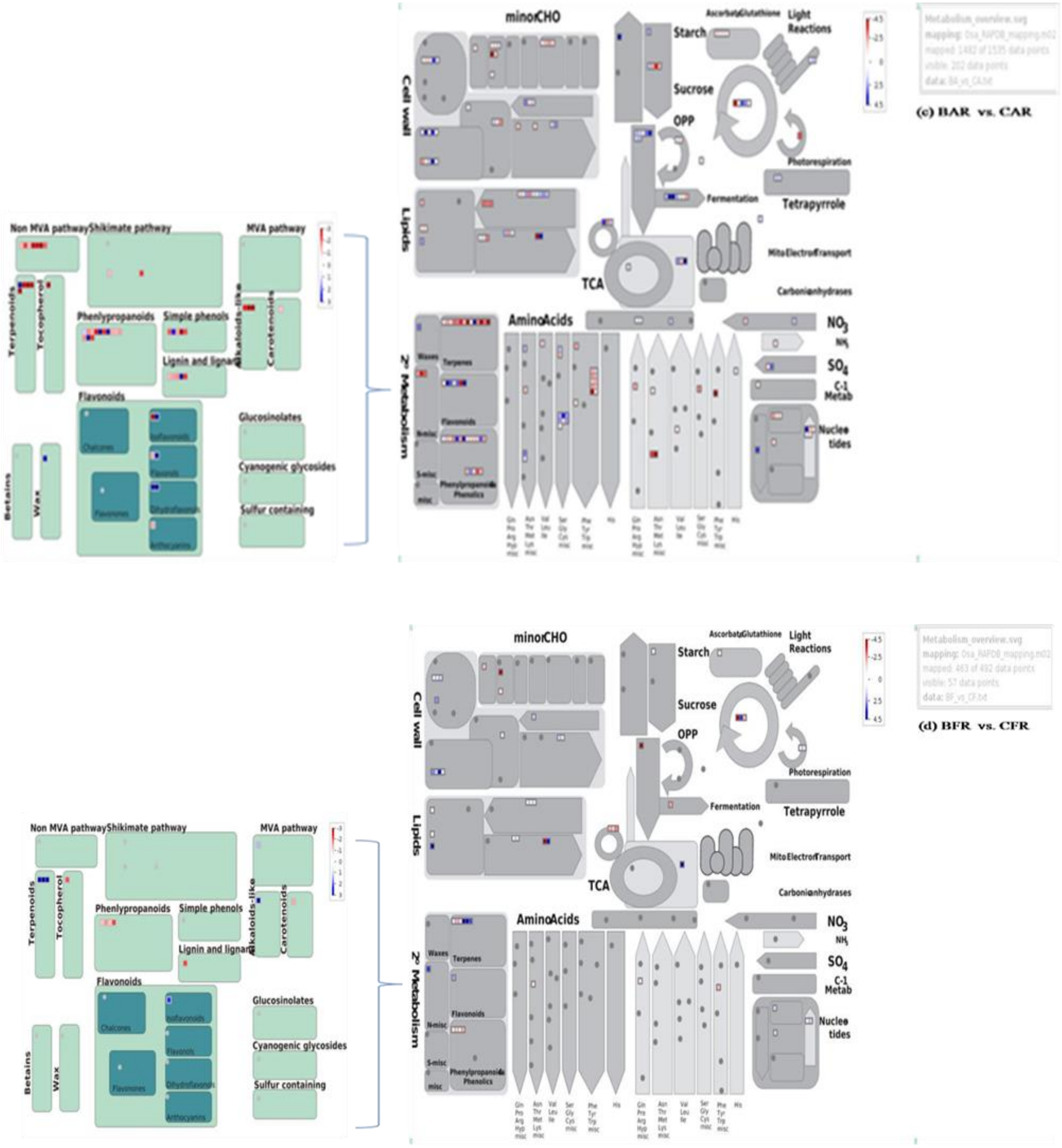

Fig. S2. Overall metabolism operating under aerobic and anaerobic conditions in shoot (a) BAS vs. CAS, (b) BFS vs. CFS and in root (c) BAR vs. CAR, (d) BFR vs. CFR.

Transcripts highlighted in insight involved in secondary metabolism processes are shown in the main panel in dark green, while putatively associated transcripts are shown in light green. Blue denotes up-regulation and red down-regulation.

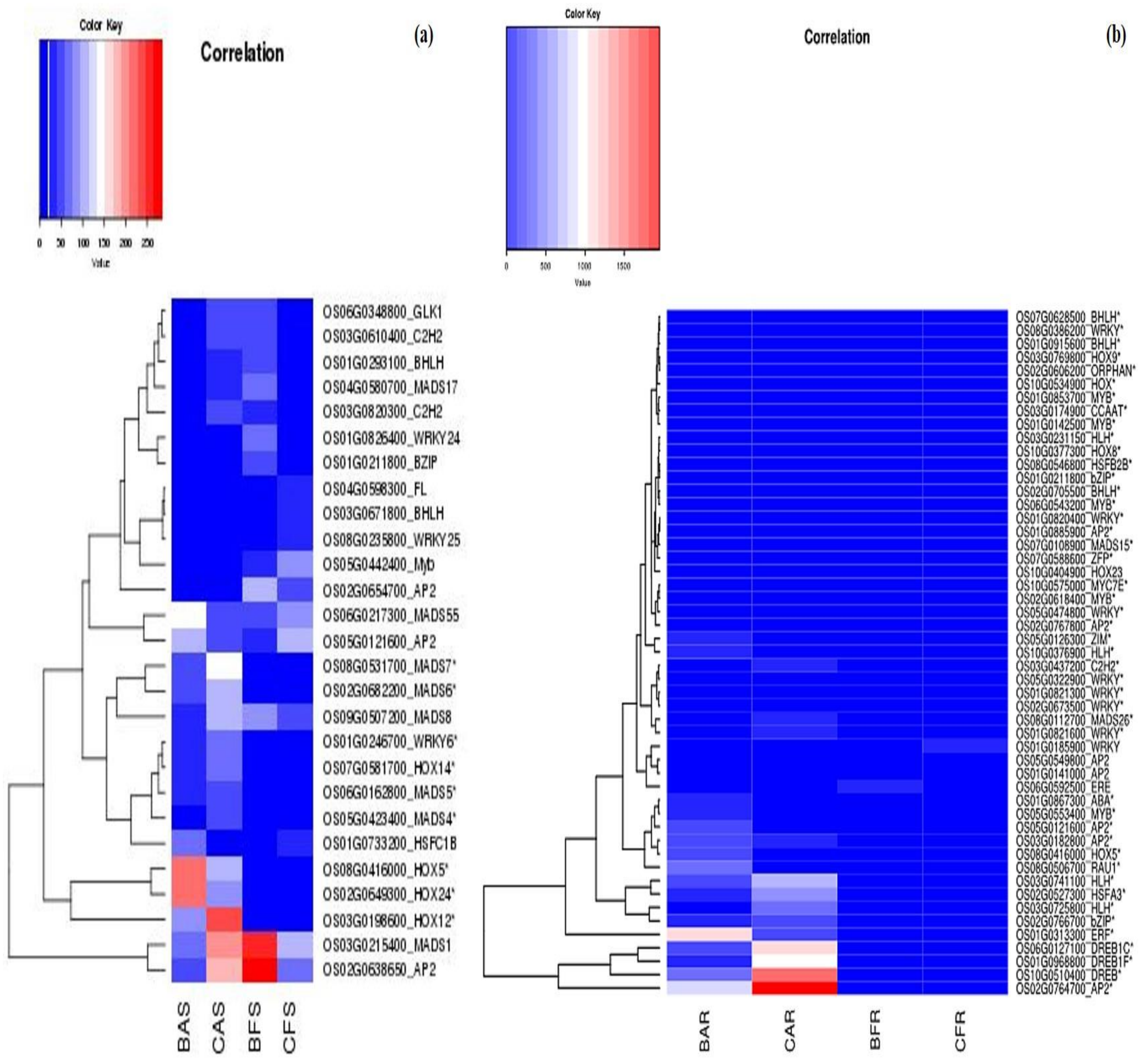

Fig. S3. Heatmaps representation of differentially expressed TFs (mentioned on the right side) (a) in shoot (BAS, CAS, BFS, CFS) and (b) in root (BAR, CAR, BFR, CFR) under aerobic and anaerobic conditions. The color scale at the top shows expression values in read count. Red represents high levels of expression and blue indicates low levels (* symbol denotes exclusively expressed TFs in shoot and root of aerobic adapted cultivar CR Dhan 202 under aerobic condition).

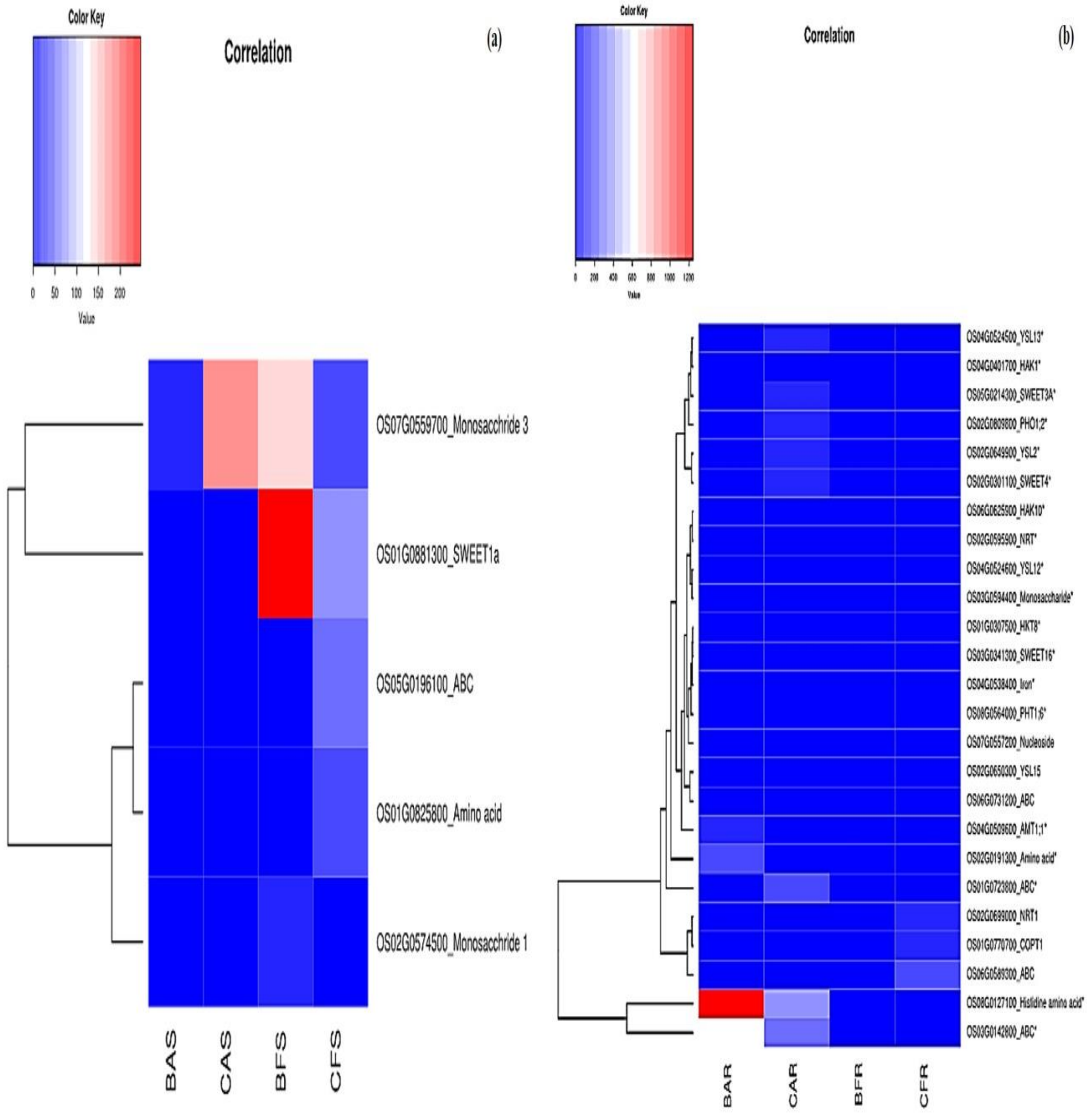

Fig. S4. Heatmaps representation of differentially expressed transporters (mentioned on the right side) (a) in shoot (BAS, CAS, BFS, CFS) and (b) in root (BAR, CAR, BFR, CFR) under aerobic and anaerobic conditions. The color scale at the top shows expression values in read count. Red represents high levels of expression and blue indicates low levels (* symbol denotes exclusively expressed transporters in shoot and root of aerobic adapted cultivar CR Dhan 202 under aerobic condition).

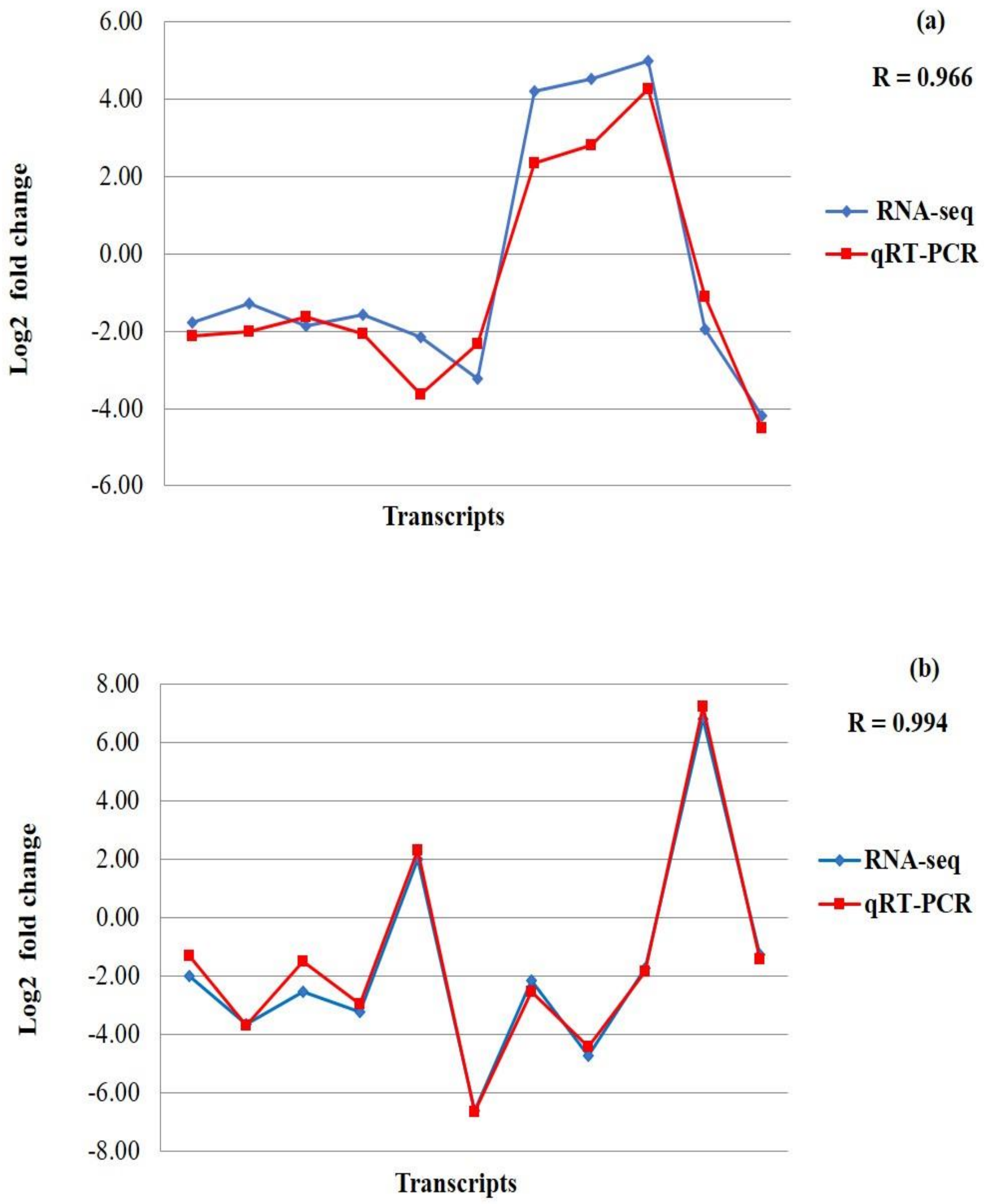

Fig. S5. Validation of the expression of representative transcripts from RNA-seq using qRT-PCR in (a) shoot (BAS vs. CAS) and in (b) root (BAR vs. CAR) under aerobic conditions.

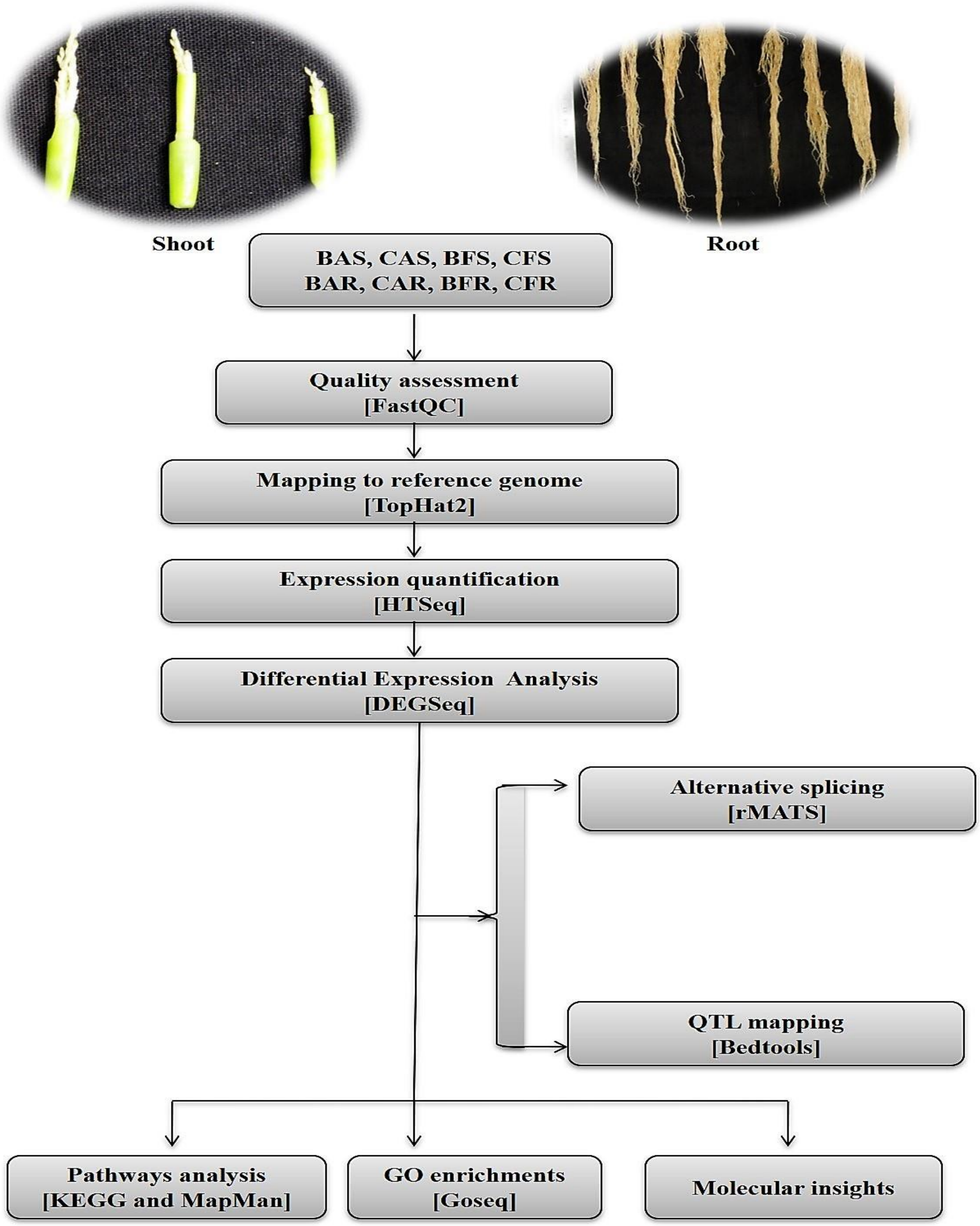

Fig. S6. Workflow/ summary of RNA-seq data analysis in shoot and root under aerobic and anaerobic conditions.

BAS - BPT 5204 Aerobic Shoot, CAS - CR Dhan 202 Aerobic Shoot, BAR - BPT 5204 Aerobic Root, CAR - CR Dhan 202 Aerobic Root, BFS - BPT 5204 Anaerobic Shoot, CFS - CR Dhan 202 Anaerobic Shoot, BFR - BPT 5204 Anaerobic Root and CFR - CR Dhan 202 Anaerobic Root

Table S1. Differentially expressed transcripts (DETs) in shoot (BAS vs. CAS) (BAF vs. CFS) and root (BAR vs. CAR) (BFR vs. CFR) under aerobic and anaerobic conditions (Please see the supplementary excel file).

Table S2. Top ten KEGG pathway enrichment analysis of the DETs of shoot and root under aerobic and anaerobic conditions.

Term	Database	ID	Input number	Background number	P-Value	Corrected P-Value
**BAS vs. CAS**						
Fatty acid degradation	KEGG PATHWAY	osa00071	5	38	0.000198021	0.006608824
Glyoxylate and dicarboxylate metabolism	KEGG PATHWAY	osa00630	6	63	0.000231889	0.006608824
Galactose metabolism	KEGG PATHWAY	osa00052	4	48	0.004134657	0.065711473
Tryptophan metabolism	KEGG PATHWAY	osa00380	3	24	0.004611331	0.065711473
Thiamine metabolism	KEGG PATHWAY	osa00730	2	9	0.008052586	0.079973496
Fatty acid metabolism	KEGG PATHWAY	osa01212	4	60	0.008632145	0.079973496
Metabolic pathways	KEGG PATHWAY	osa01100	30	1492	0.009821307	0.079973496
Plant-pathogen interaction	KEGG PATHWAY	osa04626	5	105	0.012765287	0.089848542
Peroxisome	KEGG PATHWAY	osa04146	4	70	0.014186612	0.089848542
**BFS vs. CFS**						
Metabolic pathways	KEGG PATHWAY	osa01100	63	1492	1.84334E-06	0.001198168
Biosynthesis of secondary metabolites	KEGG PATHWAY	osa01110	39	800	9.8663E-06	0.003206548
Fatty acid degradation	KEGG PATHWAY	osa00071	6	38	0.000380693	0.018963775
alpha-Linolenic acid metabolism	KEGG PATHWAY	osa00592	6	39	0.000431204	0.018963775
Terpenoid backbone biosynthesis	KEGG PATHWAY	osa00900	6	40	0.000486793	0.019775958
Cutin, suberine and wax biosynthesis	KEGG PATHWAY	osa00073	4	16	0.000845517	0.028925591
Plant-pathogen interaction	KEGG PATHWAY	osa04626	9	105	0.000940201	0.030556534
Photosynthesis - antenna proteins	KEGG PATHWAY	osa00196	4	17	0.001026452	0.031771134
Thiamine metabolism	KEGG PATHWAY	osa00730	3	9	0.002004785	0.032850376
**BAR vs. CAR**						
Biosynthesis of secondary metabolites	KEGG PATHWAY	osa01110	71	800	8.01964E-08	7.29787E-06
Metabolic pathways	KEGG	osa01100	103	1492	8.5882	0.0003101

	PATHWAY				4E-06	02
Glycolysis / Gluconeogenesis	KEGG PATHWAY	osa00010	19	125	1.0223 1E-05	0.0003101 02
Biosynthesis of amino acids	KEGG PATHWAY	osa01230	22	193	0.0001 15963	0.0026381 61
Phenylalanine, tyrosine and tryptophan biosynthesis	KEGG PATHWAY	osa00400	8	39	0.0007 0072	0.0127530 99
Phenylpropanoid biosynthesis	KEGG PATHWAY	osa00940	14	113	0.0009 35942	0.0141951 17
Carbon metabolism	KEGG PATHWAY	osa01200	22	235	0.0013 48053	0.0175246 93
Arginine and proline metabolism	KEGG PATHWAY	osa00330	7	35	0.0017 06147	0.0194074 26
Starch and sucrose metabolism	KEGG PATHWAY	osa00500	16	156	0.0025 53126	0.0258149 36
**BFR vs. CFR**						
Arachidonic acid metabolism	KEGG PATHWAY	osa00590	2	10	0.0106 99255	0.1599869 91
Plant hormone signal transduction	KEGG PATHWAY	osa04075	7	177	0.0119 14325	0.1599869 91
Biosynthesis of secondary metabolites	KEGG PATHWAY	osa01110	19	800	0.0140 62773	0.1599869 91
Pyruvate metabolism	KEGG PATHWAY	osa00620	4	72	0.0187 45371	0.1599869 91
Phenylpropanoid biosynthesis	KEGG PATHWAY	osa00940	5	113	0.0210 12259	0.1599869 91
Linoleic acid metabolism	KEGG PATHWAY	osa00591	2	15	0.0211 03488	0.1599869 91
Carbon fixation in photosynthetic organisms	KEGG PATHWAY	osa00710	4	75	0.0212 89591	0.1599869 91
Starch and sucrose metabolism	KEGG PATHWAY	osa00500	6	156	0.0216 93151	0.1599869 91
Glycolysis / Gluconeogenesis	KEGG PATHWAY	osa00010	5	125	0.0302 87904	0.1623538

Table S3. Details of uniquely expressed root related transcripts under aerobic conditions in the root of two cultivars.

Phenotype	Gene id	BAR read count	CAR read count	Gene name	Gene description
**Root development related genes**					
Crown root formation	Os01G0149800	52.8	490.9	*MT2A*	Metallothionein-like protein 2A
	Os05G0111300	150.1	657.7	*MT2C*	Metallothionein-like protein 2C
	Os01G0974200	1144	3471.7	-	Metallothionein-like protein 2B
**Genes related to physiological functions of root**					
Root hair development	Os10G0555900	6.5	34.6	*EXPB3*	Expansin-B3
**Genes related to physiological functions of root**					
P uptake	Os08G0564000	1.6	42.6	*PHT1;6*	Inorganic phosphate transporter 1-6
K and Na uptake	Os01G0307500	1.9	32.5	*HKT8*	Cation transporter HKT8
**Genes related to utilization of water and micronutrients**					
Water acquisition	Os04G0521100	2.6	22.5	*PIP2;3*	Probable aquaporin PIP2-3

Table S4. Details of alternative splicing (AS) events of differentially expressed transcripts (DETs) in shoot (BAS vs. CAS, BFS vs. CFS) and in root (BAR vs. CAR, BFR vs. CFR) under aerobic and anaerobic conditions.

Alternative Splicing (AS) Event	In shoot		In root	
	Aerobic (BAS vs. CAS)	Anaerobic (BFS vs. CFS)	Aerobic (BAR vs. CAR)	Anaerobic (BFR vs. CFR)
Retained intron (RI)	380	370	299	297
Alternative 3′ splice site (A3'SS)	170	159	138	139
Alternative 5′ splice site (A5'SS)	61	63	61	49
Skipped exon (SE)	47	46	42	42
Mutual exclusive exons (MXE)	1	1	1	0

Table S5. Details of primers used for shoot (a) and root (b) RNA-seq data validation through qRT-PCR.

S. No	Gene symbol	Gene description	Locus information	Primer sequence 5' to 3' (forward/reverse)	Amplicon length (bp)	Tm (0C)
**(a) For shoot**						
1	*OsBURP3*	BURP domain-containing protein 3	LOC_Os01g53240	CCAAGCGCAGCTCAATCTTTGC<br>TGGCTTCCTACTGAAGCGATCAAC	110	62
2	*OsCML15*	Probable calcium-binding protein CML15	LOC_Os05g31620	AAGGTGAGGGCGTTCTTCTC<br>ACCAGTCCACGTTCATCGC	91	62
3	*OsGLK1*	Probable transcription factor GLK1	LOC_Os06g24070	GCTTCAACTCCAGGTTCAACCATC<br>AGTCCAAGAGGCAAAGGCAACC	93	62
4	*OsHOX12*	Homeobox-leucine zipper protein HOX12	LOC_Os03g10210	AGCAAGCTCATGGAGGAGGAGTTC<br>TTCAGCTTCAGCAACTCGGTCTCG	131	62
5	*OsMADS8*	MADS-box transcription factor 8	LOC_Os09g32948	TCACTGATCTCCAGAGGAGGGAAC<br>CCACACTTGTCCATGCAACTGG	98	62
6	*OsMT2A*	Metallothionein-like protein 2A	LOC_Os01g05650	TGCTCGTGTGTTGTGCGATCAG<br>TCTCCGGGTACATCTTGCATCCTC	149	62
7	*OsPIP1;3*	Aquaporin PIP 1;3	LOC_Os02g57720	ACCGAGTTGATGCTGCAAGATG<br>ATCACCAGACGGTAATAACACACG	73	62
8	*OsRAB21*	Water stress-inducible protein Rab21	LOC_Os11g26790	AGCTCAAGCTCGTCTGAGGATG<br>GGGAGCTTCTCCTTGATCTTCTCC	80	62
9	*OsRAB16B*	Dehydrin Rab16B	LOC_Os11g26780	TTCGCACGTGTTGGGTTGTACC<br>ACACACACACCATACAAAGCTTGC	65	62
10	*OsTPR*	TPR Domain containing protein	LOC_Os03g19560	ACAAGCTACCCAATGCAATCCC<br>GACTTGCCTGGACTGCTTTCTC	122	62
11	*Putative*	Putative uncharacterized protein	LOC_Os05g11810	TCGTGGGAAGTTGAAGCCACTTG<br>TCTCCCGTTCGTTAGAACCTTGC	141	62
**(a) For root**						
1	*HLH*	Helix-loop-helix DNA-binding domain	LOC_Os03g51580	TCCCAGAACCAGGAACACATCC<br>CGACGCCTTGTCCATCTTCTTG	114	62

		containing protein				
2	*OsBKI1*	Brassinosteroid insensitive1-associated receptor kinase 1	LOC_Os11g31540	CATTAGCACCCTGCGAAATCTGAG<br>TCGTCAGGCTGCCCAAAGATTG	80	62
3	*OsDREB1F*	Dehydration-responsive element-binding protein 1F	LOC_Os01g73770	TTCGTCCTCGTCGGTGT<br>GCGTCTCCCGGAATTTCTT	110	62
4	*OsMT2A*	Metallothionein-like protein 2A	LOC_Os01g05650	TGCTCGTGTGTTGTGCGATCAG<br>TCTCCGGGTACATCTTGCATCCTC	149	62
5	*OsNAC71*	NAC domain-containing protein 71	LOC_Os11g08210	TTAACGGAACGTGACGCCTCTG<br>ACGATCGACCTGACAGTGGTTAG	63	62
6	*OsNAS1*	Nicotianamine synthase 1	LOC_Os03g19427	TGCTGCATTTGCGAAGCTAAGG<br>ACTGTACGTCCCAAACAACACG	76	62
7	*OsPHO1;2*	Phosphate transporter PHO1;2	LOC_Os02g56510	TAAGCCACAGCAGCCCAGAAAC<br>AGTGCCCGTGAACAAACCTACG	64	62
8	*OsPHT1;6*	Inorganic phosphate transporter 1;6	LOC_Os08g45000	TGCAAACTGTACTGTAGCGCTGTC<br>TGGCGGTTTCTTCGATCGATCTTC	124	62
9	*OsPIP1;3*	Aquaporin PIP 1;3	LOC_Os02g57720	ACCGAGTTGATGCTGCAAGATG<br>ATCACCAGACGGTAATAACACACG	73	62
10	*OsRAB21*	Water stress-inducible protein Rab21	LOC_Os11g26790	AGCTCAAGCTCGTCTGAGGATG<br>GGGAGCTTCTCCTTGATCTTCTCC	80	62
11	*OsYSL2*	Metal-nicotianamine transporter YSL2	LOC_Os02g43370	ACTTGCTTCCACTCAGAAAGGC<br>GTTGCAGTCCCACTTGGATAGG	67	62
